# Immune-enhancing effect of fermented soybean food, *Cheonggukjang* on cyclophosphamide-treated immunosuppressed rat

**DOI:** 10.1016/j.heliyon.2024.e37845

**Published:** 2024-09-14

**Authors:** Hak Yong Lee, Young Mi Park, Dong Yeop Shin, Hai Min Hwang, Han Na Jeong, Hyo Yeon Park, Hee-Jong Yang, Gwang Su Ha, Myeong Seon Ryu, Ji Won Seo, Do-Youn Jeong, Jun Sang Bae, Byeong Soo Kim, Jae Gon Kim

**Affiliations:** a INVIVO Co. Ltd., 121, Deahak-ro, Nonsan, Chungnam, 32992, Republic of Korea; b Microbial Institute for Fermentation Industry (MIFI), Sunchang, 56048, Republic of Korea; c Department of Integrated Life Science and Technology, Kongju National University, 32439, Republic of Korea; d Department of Pathology, College of Korean Medicine, Wonkwang University, Iksan, Republic of Korea

**Keywords:** *Cheonggukjang*, Immune-enhancing, Cytokines, Inflammatory pathways, Spleen

## Abstract

Cheonggukjang (*CGJ*) is a traditional food, made by the fermentation of beans, and it has different recipes for different regions in Korea. However, it has anti-inflammatory, anti-cancer, and anti-obesity effects, and is known to affect changes in the intestinal microbiota. In this study, we investigated the immune-enhancing effects of four type *CGJ*s (one commercial and three transitional *CGJ*s). In the cyclophosphamide (CP)-treated immunosuppressed rat, oral administration of *CGJ*s for 4 weeks was used to investigate weight of body and immune organ, change of microbiota, blood and serum parameters, inflammation pathways (MAPKs and NFκB) and histology of spleen. It showed an immunity-enhancing effect through increase Bacteroidetes in gut, the recovery of complete blood count, levels of cytokines and IgG, activation of inflammatory pathways, and histology of spleen. In conclusion, these results show that the intake of a commercial brand CGJ, and traditional CGJs can maintain or promote the body's immunity.

## Highlights

• *Cheonggukjang* regulate *Firmicutes*/*Bacteroidetes* ratio.
• *Cheonggukjang* promotes the IL-2, IL-12, IFN-γ and IgG productions in immunosuppression status.
• *Cheonggukjang* increases proliferation of splenocyte and NK cell activity.
• *Cheonggukjang* activate MAPKs and NFκB pathways.

## Graphical abstract

**Figure:**
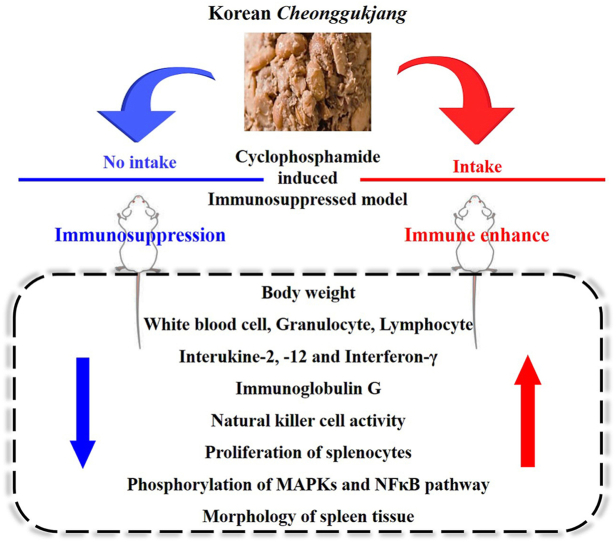

## 1 Introduction

The immune-enhancing effect restores the decline in the body's immunity due to various factors such as viruses, stress, aging, insomnia, and immunosuppressive agents [[1], [2], [3]]. Decreased immunity is a factor that causes various immune diseases, as it becomes impossible to defend against harmful substances [4]. Recently, amid the coronavirus disease (COVID-19) pandemic, there has been a growing interest in immunity and the intake of foods, health-functional foods, and nutrients known to enhance immunity [5,6]. Representative immune-enhancing materials include red ginseng and nutritional supplements (including Zn), and many studies have shown that the ingredients of natural products, fruits, and probiotics improve immunity in mammals [[7], [8], [9], [10], [11], [12]]. The fermentation of natural products benefits health by increasing their beneficial nutrients or creating new beneficial substances [13].

Fermentation (or bioconversion) refers to the phenomenon in which microorganisms or *Latobacillus* (such as *Latobacillus plantarum*, *Latobacillus reuteri*, and *Lactobacillus rhamnosus*) are mixed, decomposed, and changed [[14], [15], [16]]. Fermentation technologies are used worldwide. Fermentation methods include alcohol, lactic acid and acetic acid of fermentation, protein decomposition, and glycolysis and are applied to the fermentation of food [17]. Fermented foods include kimchi, yogurt, cheese, vinegar, doenjang (fermented soybean paste), and gochujang [18,19]. Several recent studies have investigated the efficacy of fermented foods. In fermented foods, anthocyanin and flavonoid components increase, beneficial bacteria increase, and harmful bacteria decrease, which are reported to be effective against cardiovascular diseases, obesity, intestinal health, blood flow improvement, anti-cancer effects, and antidiabetic effects [13,20,21].

*Cheonggukjang* (*CGJ*) is a representative food fermented with beans and *Bacillus subtilis* [22], and it is a fermented food that contains beneficial ingredients such as probiotics, isoflavones, and subtilin, which are known to help prevent various diseases, and amino acids (such as leucine, isoleucine, lysine, and tryptophan), fatty acids, and proteins. Its production is made in two ways by commercial (standardized recipe) or traditional recipes (different recipes for each person) and it is known to have different taste and aroma due to the difference in fermentation conditions due to differences in temperature and humidity depending on the region being made [23]. And it has been reported to play a role in physiological activities, including antioxidant, anti-inflammatory, anti-cancer, thrombolysis, prevention of hypertension and osteoporosis, and reduction of blood cholesterol [24,25]. In the previous studies, it have reported improvements in memory through brain inflammation and antidiabetic effects, improvement in intestinal inflammation in dextran sulfate sodium (DSS)-induced colitis, reduction in fatty liver and body weight in a high-fat diet model, and water extract from *CGJ* (fermented by *Bacillus amyloliquefaciens*, SRCM100730) and polysaccharides from *CGJ* increased immune enhancement and immune regulation through activation of the NF-κB pathway in RAW 264.7 macrophages and cyclophosphamide (CP)-induced immunosuppression [[26], [27], [28], [29], [30]]. However, research results on the efficacy of *CGJ*s made in various regions are insufficient, and there are no research results on the difference between commercial *CGJ* and its efficacy.

In this study, the intake of one commercial brand *CGJ* and three traditional *CGJ*s (other regions in Korea) in CP-induced immunosuppressed rats for 4 weeks was used to investigate the immunity enhancement effects through body weight, immune tissue weight, change of microbiomes (before and after intake), immune cell in whole blood, cytokine secretion, proliferation of splenocyte, natural killer cell activity and histological analysis.

## 2 Materials and methods

### 2.1 Preparation of Cheonggukjang (CGJ)

The four types of *CGJ* were provided by the Microbial Institute for Fermentation Industry (MIFI, Jeollabuk-do, Korea). Information on *CGJ*s were as follows: S1 was a commercial brand (Salt-free *CGJ*, Agricultural corporation Sunchang, Sunchang-gun, Jeollabuk-do, Korea); *CGJ*; S2 was Iksan-si (Jeollabuk-do, Korea); S3 was Sunchang-gun (Jeollabuk-do, Korea); and S4 was Pocheon-si (Gyeonggi-do, Korea). *CGJ*s (S2-S4) was traditional made in the recipe of each region. *CGJ*s was finely ground using a blender for 60 s in distilled water (DW; 1:1). After drying, the weight of the dried product was measured (S1: 227.9 ± 1.4 mg/mL, S2: 191.5 ± 1.6 mg/mL, S3: 255.9 ± 2.1 mg/mL, S4: 229.4 ± 0.7 mg/mL), and oral administration was performed daily at 500 mg/kg based on the dry weight.

### 2.2 Chemical, biogenic amines and aflatoxin analysis of CGJs

Chemical analysis of *CGJ*s (S1-S4) was determined using a previously reported method (the Korean Ministry of Food and Drug Safety disclosed Notification No. 2021-54 in Food Code) [31]. Using Kjeldahl method measured the crude protein content and crude fat content was determined using the Soxhlet method. Calories were calculated by Atwater's coefficient. The Biogenic amines (histamine and tyramine) and aflatoxins were assessed as previous our study [31]. It was analyzed using High Performance Liquid Chromatography (HPLC, Agilent 1200 series) equipped with Capcellpak C18 column for biogenic amines, and Shiseido UC 120 column for aflatoxin.

### 2.3 Animal and oral administration

All the experiments were conducted following the National Institutes of Health Guidelines for the Care and Use of Animals. This study was approved by the Institutional Animal Care and Use Committee of INVIVO Co., Ltd. (IV-RB-17-2305-15). Wistar rats (5 weeks old, male) were purchased from Orient BIO Co. (Gyeonggi-do, Korea). Wistar rats were divided into seven groups (normal, control, S1, S2, S3, S4, and positive group). Immunosuppressed rats were orally administered CP (5 mg/kg). Wistar rats were orally administered *CGJ*s (500 mg/kg) or HemoHIM, herbal preparation (1,000 mg/kg, positive group; purchased from Kolmar BNH Co., Ltd., Sejong, Korea) with CP for 4 weeks. HemoHIM used in the positive group was used because it was known to have immune-enhancing effect [32]. The administration dose was established by referring to previous studies [28,33,34]. All animals were monitored weekly for body weight during the experimental period. And At autopsy, weights of thymus and spleen tissue were immediately measured.

### 2.4 Intestinal microbial analysis

Intestinal microbial analysis of *CGJ* was performed using Next-Generation Sequencing as described previously [34]. Briefly, the DNA from stool of S1-S4 groups was extracted by DNeasy PowerSoil Kit (Qiagen, Germany), and amplified with V3-V4 regions of the targeting primers. Sequencing was performed on the Illumina Miseq platform at the MIFI. Sequences were taxonomically classified at different levels (phylum and genus). The distribution of microorganisms before and after intake of *CGJ*s was performed by principal coordinates analysis (PCoA) with Bray-Curtis distance metrics [35].

### 2.5 Complete blood cell (CBC) count analysis

After respiratory anesthesia, whole blood was collected from the vena cava and kept ethylenediaminetetraacetic acid (EDTA)-coated tube. Immediately, count of CBC was measured by Mindray BC-2800 hematological analyzer (Mindray, Bath, UK).

### 2.6 Cytokine and immunoglobulin G levels in serum

Whole blood from vena cava kept in conical tube (without EDTA). It was coagulated at room temperature for 30 min and serum was separated in a centrifuge at 3,000 rpm for 15 min at 4 °C. The levels of interleukin (IL)-2, interferon (IFN)-γ, tumor necrosis factor (TNF)-α, and IgG in serum were measured using enzyme-linked immunosorbent assay (ELISA) kit (IL-2, MBS269718, Mybiosource; IFN-γ, ab239425, Abcam; TNF-α, CSB-E11987r, CUSABIO; IgG, ab189578, Abcam). The results were measured using a Sunrise™ ELISA plate reader (Tecan, Männedorf, Switzerland).

### 2.7 Primary cell culture and cell proliferation in splenocyte

Splenocytes from Wistar rat was cultured in following method. Spleen tissues gently crushed by needles and then though a cell strainer (70 μm, SPL Life Sciences, Gyeonggi-do, Korea). The collected splenocytes were washed in RPMI-1640 (Invitrogen, Ca, USA). And red blood cell was removed by red blood cell lysis buffer (Sigma-Aldrich, MO, USA). Splenocytes were maintained in RPMI-1640 with 10 % fetal bovine serum (FBS) and 1 % penicillin-streptomycin (10,000 U/mL, Invitrogen, Ca, USA) in 5 % CO_2_ incubator. After 24 h stabilization of splenocytes (5 × 10^5^ cells/well), cell proliferation was measured by WST-1 Assay Kit (ITSBio, Seoul, Korea) and ELISA plate reader.

### 2.8 Natural killer (NK) cell activity

For the NK cell activity, target cell, AR42J cell (CRL-1492) purchased from the ATCC (VA, USA). And primary splenocytes were isolated from all groups (normal, control, *CGJ*-treated, and positive) for use as effector cells. These cells were co-cultured in 96-well plates at a ratio of effector cells to target cells (25:1) in 5 % CO_2_ incubator at 37 °C for 24 h AR42J viability rate was measured by WST-1 Assay Kit and ELISA plate reader. NK cell activity was calculated as the survival rate of AR42J cells compared to the normal group.

### 2.9 Tissue lysis and western blot analysis

For spleen tissue disruption, it was lysed with PRO-PREP™ Protein Extraction Solution (Cat No. 17081, iNtRON) containing complete protease inhibitor cocktail (Roche, Basel, Switzerland). Total protein (10 μg/well) was separated with 10 % sodium dodecyl sulfate-polyacrylamide gel electrophoresis, followed by transfer to PVDF membranes (BIO-RAD, CA, USA) and blotted with the indicated specific antibody. Specific antibodies such as Phospho-Erk (#9101), Erk (#9102), Phospho-p38 (#9216), p38 (#9212), Phospho-JNK (#4671), JNK (#9252), Phospho-NFκB (#3033), NFκB (#8242), and β-actin (#4857) purchased from the Cell Signaling Technology (MA, USA). Target band intensity was detected using a Western blot imaging system (Azure Biosystems c300) and quantified by using ImageJ software (NIH, MD, USA).

### 2.10 Spleen histological analysis

Briefly, the spleen separated from Wistar rat (all group) was weighed and fixed in 10 % formalin. After 48 h, fixed tissues were embedded in paraffin and sectioned slices (4 μm) using a microtome (Thermo Scientific, MA, USA). The sectioned spleens were stained using hematoxylin and eosin (H&E). Spleen images were scanned using Motic EasyScan One (Motic, Hong Kong).

### 2.11 Statistical analysis

All data are expressed as the mean ± standard error of the mean (SEM), and differences between groups were analyzed using one-way ANOVA (Duncan's multiple-range test). All analyses were performed using SPSS version 23.0 (SPSS Inc., USA). Each value represents the mean of at least three independent experiments for each group. Statistical significance was set at *p* < 0.05.

## 3 Results

### 3.1 Components and chemical analysis

We analyzed the ingredients of *CGJ*s (S1–S4). Although the baseline for biogenic amines (BAs) for Jangs such as *Gochujang*, *Doenjang* (fermented soybean paste), *Ganjang* (soy sauce), and *CGJ* has not yet been established in South Korea, *CGJ*s (S1–S4) was investigated within the reference range when compared with fishery products from the European Union (EU, total BAs 300 mg/kg) and United States Food and Drug Administration (US FDA, total BAs 1,000 mg/kg) (Table 1) [36,37]. And total aflatoxin was not detected in *CGJ*s (S1–S3) but *CGJ* S4 (0.26 ± 0.10 μg/kg). And microbial distribution (%) was low the content of harmful bacteria cause food poisoning (Table 1). In Table 2, the chemical analysis (such as water content, sodium content, carbohydrate, crude protein, crude fat, calories, and dietary fiber) of *CGJ*s (S1–S4) were analyzed. In these results show that it was investigated that there are differences in BAs, microbial distribution, and chemical ingredient on other regions in South Korea.

**Table 1:** Information and component of *Cheonggukjang*.

Cheonggukjang	Region (in Korea)	Biogeni camine (mg/kg)		Total Aflatoxin (μg/kg)	Microbial distribution (%)	
		Histamine	Tyramine		Beneficial bacteria	Harmful bacteria
S1	Sunchang-gun, Jeollabuk-do	0.20 ± 0.17	3.84 ± 1.20	N.D	25.94 ± 0.44	0.09 ± 0.00
S2	Iksan-si, Jeollabuk-do	2.17 ± 0.07	5.70 ± 1.34	N.D	71.54 ± 2.41	2.68 ± 0.44
S3	Sunchang-gun, Jeollabuk-do	2.37 ± 0.64	228.49 ± 11.17	N.D	4.75 ± 0.42	N.D
S4	Pocheon-si, Gyeonggi-do	1.30 ± 0.28	0.30 ± 0.31	0.26 ± 0.10	94.56 ± 1.22	0.02 ± 0.00

N.D: Not detection.

**Table 2:** Chemical analysis of *Cheonggukjang*.

Cheonggukjang	Water content (%)	Sodium content (mg/100 g)	Carbohydrate (g/100 g)	Crude Protein (g/100 g)	Crude Fat (g/100 g)	Calorie (kcal/100 g)	Dietary fiber (g/100 g)
S1	52.47 ± 0.56	N.D	20.11 ± 0.22	21.36 ± 0.67	1.75 ± 0.42	147.38 ± 1.83	16.90 ± 1.24
S2	59.37 ± 0.23	0.68 ± 0.10	16.22 ± 0.85	18.19 ± 0.14	2.35 ± 0.12	129.89 ± 1.12	14.45 ± 4.48
S3	49.32 ± 0.74	1.72 ± 0.04	18.98 ± 0.42	21.65 ± 0.48	3.10 ± 0.28	159.6 ± 2.02	15.41 ± 2.45
S4	52.38 ± 0.56	0.07 ± 0.04	21.11 ± 0.65	20.04 ± 0.55	2.26 ± 0.36	142.94 ± 0.73	21.00 ± 1.97

N.D: Not detection.

### 3.2 Body, immue-related tissues weight and stool microbiom monitoring

**Fig. 1:**
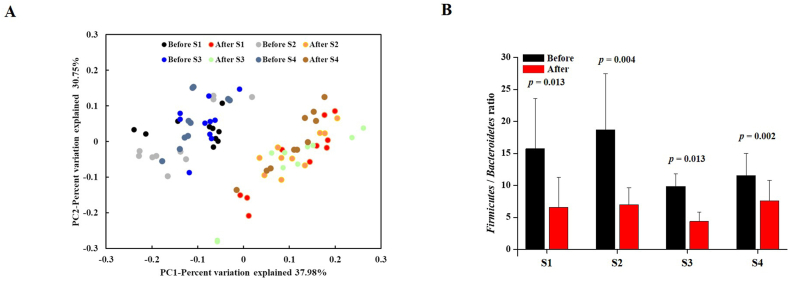
Comparison of the gut microbiota. (A) Principal coordinate analysis (PCoA) plot and (B) *Firmicutes*/*Bacteroidetes* ratio analysis results of microbial communities at the species level based on Bray-Curtis.

**Suppl. Fig. 1:**
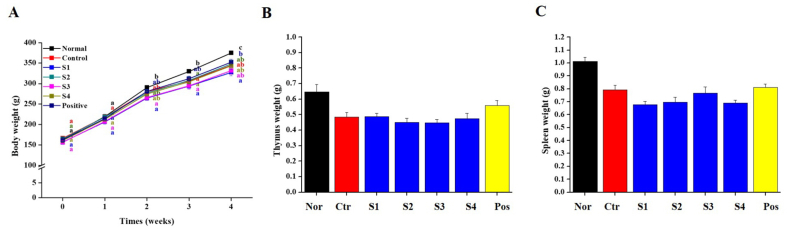
Effect of CGJs (S1-S4) on weight of body weight and immune organs (thymus and spleen) in Cyclophosphamide (CP)-induced immunosuppressed Wistar rats. Bars labeled with different superscripts have significantly different values (p < 0.05).

**Suppl. Fig. 2:**
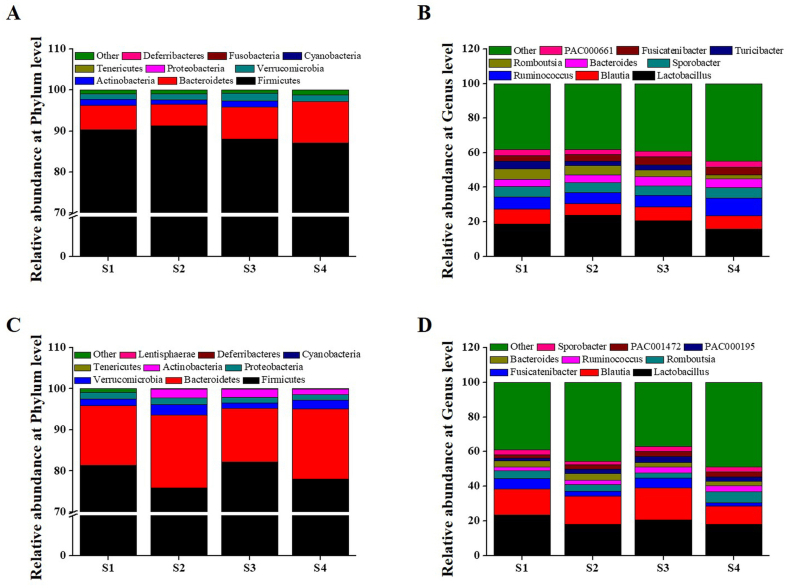
Microbial composition in the CGJs (S1–S4): Relative abundance (%) at (A) the phylum level of before intake; (B) the genus level of before intake; (C) the phylum level of after intake; and (D) the genus level of after intake.

We investigated the immune-enhancing efficacy of orally administered *CGJ*s (S1–S4, 500 mg/kg) in CP (5 mg/kg)-treated immunosuppressed rats. First, we measured body weight (BW) changes during immunosuppression by CP and after 4 weeks of oral administration of *CGJ*s (S1–S4). At 4 weeks, in the control group (only CP group, 344.90 ± 4.86 g), BW was reduced compared to that in the non-treated group (normal group, 374.97 ± 4.76 g). Moreover, oral administration of *CGJ* (S1, 327.4 ± 8.19 g; S2, 348.14 ± 9.07 g; S3, 331.66 ± 8.9 g; S4, 343.09 ± 6.64 g) in CP groups did not affect the change in BW compared to that in the control group. The weight in the positive control group, the HemoHIM (1,000 mg/kg)-treated group (352.53 ± 7.23 g), significantly increased compared to that in the control group (Suppl. Fig. 1A). Additionally, the weight of immune-related tissues (thymus and spleen) was compared, but there was no difference (Suppl. Figs. 1B and C). Secondly, at autopsy, we measured the weight of immune related tissues (thymus and spleen) in all experimental groups. Thymus and spleen weights in the CP-treated group were lower than those in the normal group. The thymus and spleen weights of the S1–S4 groups did not differ from those of the control group. Lastly, we compared the changes in intestinal microorganisms in the stool samples before and after intake of *CGJ*s (S1–S4; Fig. 1 and Suppl Fig. 2). To explore if differences in microbiome composition correlate, we computed the between-with before and after intake of *CGJ*s using Bray-Curtis distance (Fig. 1A). Through PCoA analysis, we showed that the gut microbiota was changed by the intake of *CGJ*s. At the phylum level, before the intake of *CGJ*s, *Firmicutes* were the most abundant, and its distribution was significantly reduced after intake. In contrast, *Bacteroidetes* showed a small distribution before intake, and a significant increase was observed after the intake of *CGJ*s (Suppl. Fig. 2). In the *Firmicutes*/*Bacteroidetes* (F/B) ratio, compared to the before intake of F/B ratio, the after intake of F/B ratio was analyzed to be significantly decreased (Fig. 1B). These results show that the intake of *CGJ*s did not affect the BW and immune-related tissues weight, and increased *Bacteroidetes* and decreased *Firmicutes* in phylum at stool in the CP-treated immunosuppressed rats.

### 3.3 Complete blood count, cytokines and immunoglobulin G level

**Fig. 2:**
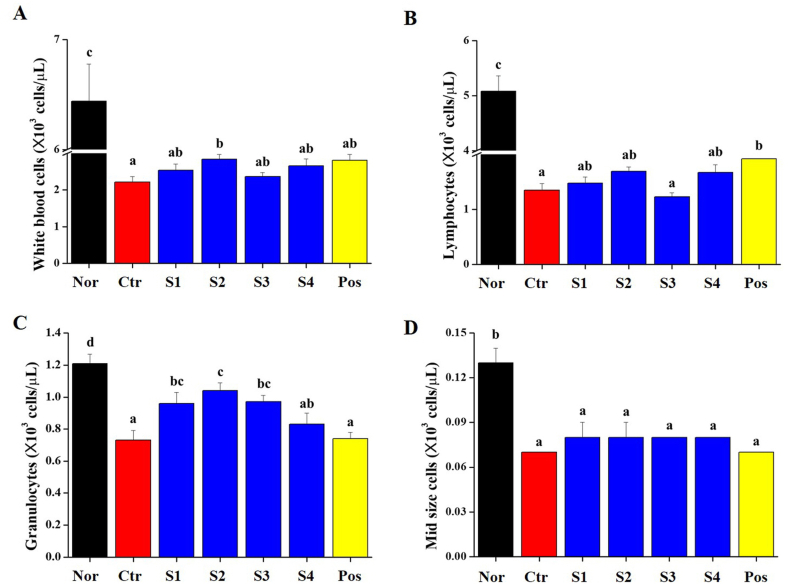
Effects of *CGJ*s (S1-S4) on (A) white blood cell; (B) granulocyte; (C) lymphocyte; and (D) mid-size cells absolute counts in the whole blood of Cyclophosphamide (CP)-induced immunosuppressed Wistar rats. Bars labeled with different superscripts have significantly different values (*p* < 0.05).

**Fig. 3:**
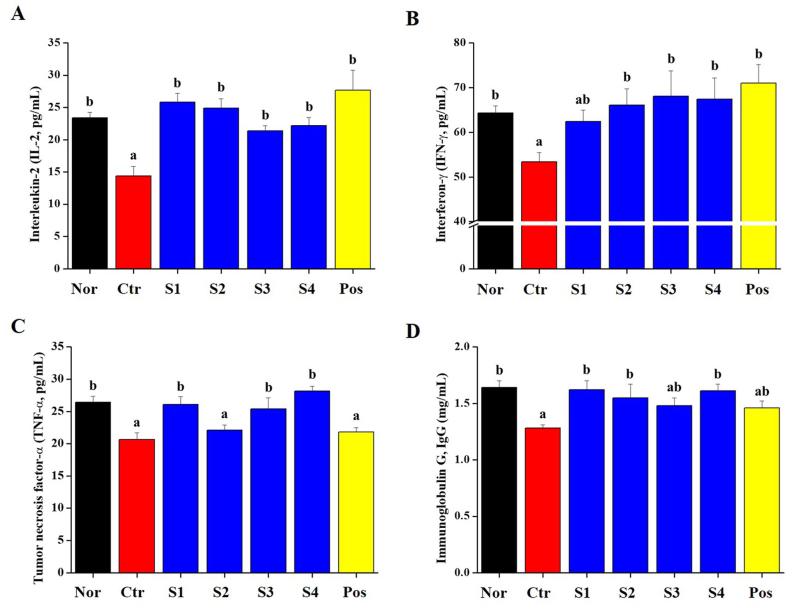
Effect of *CGJ*s (S1-S4) on the serum levels of immune-related cytokines in the serum of Cyclophosphamide (CP)-induced immunosuppressed Wistar rats. Serum levels of (A) IL-2; (B) IFN-γ; (C) TNF-α; and (D) IgG were quantified using ELISA kits. Bars labeled with different superscripts have significantly different values (*p* < 0.05).

Immune regulation and responses are mediated by immune cells (such as WBCs, granulocytes, lymphocytes, and macrophages), cytokines and immunoglobulins. Whole blood was collected from the vena cava, followed by the analysis of WBC, granulocytes, lymphocytes, and mid-sized cells (Fig. 2). The number of WBC in the CP-treated group was significantly reduced, and all groups that consumed *CGJ*s (S1–S4)-treated groups showed increased in whole blood (Fig. 2A). Granulocytes in the WBC were significantly reduced in the CP-treated group and significantly increased in the *CGJ*s (S1–S4) groups (Fig. 2B). In lymphocytes, it was increased in the S1, S2, and S4 groups but not in the S3 group (Fig. 2C). Finally, the mid-group did not differ from the control group (Fig. 2D). In the serum, we investigated the level of inflammatory cytokines (such as IL-2, INF-γ, and TNF-α) (Fig. 3A–C). In addition, IgG levels were measured as immune markers (Fig. 3D). In the CP-treated group, the level of IL-2, INF-γ, TNF-α, and IgG in serum significantly decreased. In *CGJ*s (S1–S4), the level of cytokines (IL-2, INF-γ, and TNF-α) and IgG showed a normal level. In the S2-treated group, the level of TNF-α did not protect the level of reduction by CP (Fig. 3C). These results indicated that the intake of *CGJ*s (S1–S4) suppressed decrease of immune cells, cytokines and immunoglobulin G by CP.

### 3.4 Proliferation of splenocyte and natural killer (NK) cell activity

**Fig. 4:**
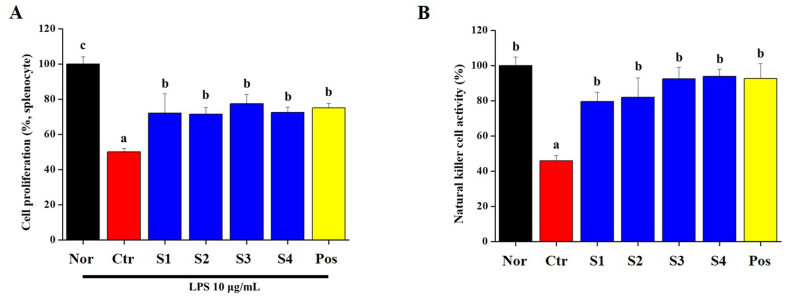
Effect of *CGJ*s (S1-S4) on (A) proliferation of splenocyte with LPS (10 μg/mL) and (B) natural killer (NK) cell activity from Cyclophosphamide (CP)-induced immunosuppressed Wistar rats. Bars labeled with different superscripts have significantly different values (*p* < 0.05).

The spleen is responsible for the protective immune response to antigens and is the main lymphatic organ in which lymphocyte differentiation occurs. Splenocyte proliferation is important in the immune system. Splenocytes were isolated from the normal, CP-treated, *CGJ*s (S1–S4)-treated, and positive groups. Splenocyte proliferation was investigated in the presence of LPS (Fig. 4A). The proliferation of splenocytes isolated from the CP-treated group was significantly reduced compared to that of the normal group. In addition, the proliferation of splenocytes in the *CGJ*s (S1–S4)-treated and positive groups increased significantly compared to that in the CP-treated group (Fig. 4A). NK cell activity is an indicator of immunity, and we investigated NK cell activity in splenocytes from Wistar rats in all groups (Fig. 4B). Lactate dehydrogenase (LDH) production was evaluated in effector (splenocytes) and target cells (AR42J cells). NK cell activity (%) was significantly lower in the CP-treated group than in the normal group. In contrast, it was significantly increased by oral administration of *CGJ*s (S1–S4) and HemoHIM (positive). These results suggest that *CGJ*s enhance immunity by increasing splenocyte proliferation and NK cell activity in the spleen.

### 3.5 Inflammation pathways

**Fig. 5:**
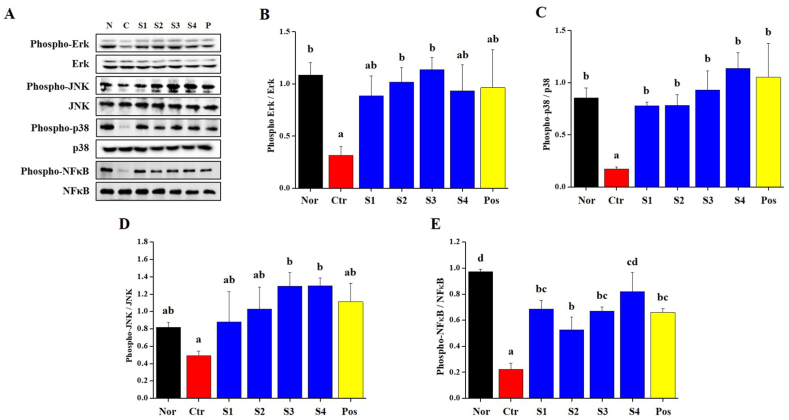
Anti-inflammatory related signal pathways on splenocyte from Cyclophosphamide (CP)-induced immunosuppressed Wistar rats. (A) Representative image of anti-inflammatory related protein expression and signal pathway (MAPKs, and NFκB); (B) Erk; (C) p38; (D) JNK; and (E) NFκB determined with Western blot analysis. Bars labeled with different superscripts have significantly different values (*p* < 0.05).

Next, we analyzed the phosphorylation of MAPKs (Erk, JNK, and p38) and NFκB at spleen tissue in regulate immune mechanisms (Fig. 5). CP, an immunosuppressant, has been observed to inhibit the phosphorylation of MAPKs and NFκB. Phosphorylation of MAPKs and NFκB inhibited by CP in the spleens of *CGJ*s (S1–S4) groups was increased to normal group levels (Fig. 5B–E). This result was explained that the intake of *CGJ*s induces activation of immune-related mechanisms in the spleen, thereby affecting immunity.

### 3.6 Histological assay

**Fig. 6:**
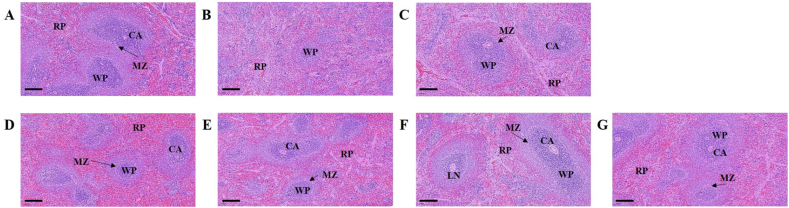
Effect of *CGJ*s (S1-S4) on immunity-associated spleen damage in Cyclophosphamide (CP)-induced immunosuppressed Wistar rats. Representative images of the sectioned spleens of (A) normal rats (saline); (B) control rats (only CP); (C) positive control (treated with Cy and 1,000 mg/kg of HemoHIM); (D) S1; (E) S2; (F) S3; (G) S4. Scale bar = 500 μm. CA, central artery; LN, lymph nodule; MZ, marginal zone; RP, red pulp; WP, white pulp.

Finally, lesions were observed in the spleen tissues of all groups. In the spleen tissue of normal group, white pulp (WP) surrounds the central artery, and the lymph nodes is at the edge, so the boundary with red pulp (RP) was clearly observed (Fig. 6A). In contrast, the CP-treated group showed contraction of WP, disruption of marginal zone (MZ), and lymphoid depletion in spleen tissue (Fig. 6B). In the spleen tissues of *CGJ*s (S1–S4)-treated groups and positive group improved contraction of WP, disruption of marginal zone (MZ), and lymphoid depletion compared to the CP-treated group. However, in the S1- and S2-treasted groups, MZ was not clearly observed. S3- and S4-treated groups were similar to those of the normal group. These results suggest that the intake of *CGJ*s can improve immunity by suppressing the collapse of the immune system.

## 4 Discussion

Fermented food, *CGJ*s (S1-S4) from one commercial brand and three different regions of South Korea were investigated for their immune-enhancing effects. In this study, we observed immune-related indicators that change due to the intake of *CGJ*s. Our results show the intake of *CGJ*s show and effect on change of microbiomes at stool, secretion of cytokines (IL-2, IFN-γ, and TNF-α), and IgG levels in serum, splenocyte proliferation, NK cell activity, and histology of spleen. However, it did not affect on BW and immune tissue (thymus and spleen) weight (Supplementary Fig. 1). These results suggest that the intake of *CGJ*s has an immune-enhancing effect by help to activated the immune system.

In decreased immunity, it is reported that the reduction of BW and immune tissues (thymus and spleen) were observed in CP-treated immunosuppressed animal model [32]. BW was measured weekly as an external factor, and reduction of weight was observed by CP (Suppl. Fig. 1A). After the end of experiment, immune tissues were immediately measured. Weight of thymus and spleen were also reduced by CP (Suppl. Figs. 1B and C). Intake of *CGJ*s, it was investigated that it did not affect the improvement of the weight of BW and immune tissues. However, the commercial brand *CGJ* (S1) was significantly improved BW. These results were explained that traditional *CGJ* (S2-S4) does not affect BW and weight of immune tissues.

The gut microbiota has been reported to be helpful for the intestinal health, furthermore, also brain health, skin health, and immunity [38]. *Bacteroidetes* and *Firmicutes* are representative in the gut microbiota and occupy a large distribution. *Bacteroidetes* the important regulators of gut health such as inflammation, gastrointestinal barrier homeostasis, and maintain immune-function [39,40]. Intake of probiotics and fermented foods (such as *Gochujang*, *Doenjang*, *Kimchi*, and cheeses etc.) induces changes in the gut microbiota. In the previous study, intake of probiotics, Live *Lactobacillus sakei* K040706 is reported that its induces changed in the intestinal microbiota (increased *Bacteroidetes* and decreased *Firmicutes*), increased proliferation of T and B cell by LPS and concanavalin A, decreased the expression of pro-inflammatory mediator (such as NF-κB, signal transducer and activator of transcription 3 and toll-like receptor 4), increased NK cell activity, and alleviates DSS-induced colitis and affects the strengthening of the intestinal wall [41,42]. Additional, the intake of fermented natural products explained that it overcome immune diseases such as COVID-19 [43]. The *CGJ*s has also been reported to contain many beneficial bacteria as fermentation products of soybeans [44]. Therefore, we showed a comparison of before and after intake of *CGJ*s (Fig. 1 and Suppl. Fig. 2). In our result, the intake of *CGJ*s for 4-week revealed that it induces changes of increase in *Bacteroidetes* and decrease in *Firmicutes* through PCoA analysis and F/B ratio (Fig. 1). *Bacteroidetes* is known to increase when abundant dietary fiber enters the intestine [45]. Therefore, it seems to have increased due to the intake of *CGJ*s, which has a lot of dietary fiber (Table 2). It is known that when increased *Firmicutes* and decreased *Bacteroidetes*, occurred obesity and colitis [46,47]. The decreased *Firmicute* by *CGJ* due to suggests prevention of obesity and colitis. Therefore, in our result, increased *Bacteroidetes* and decreased *Firmicutes* suggested that the intake of *CGJ*s induces changes in the gut microbiota and may be involved in the immunity.

Immunity is activated by immune cells and cytokines. These cells are involved in innate immunity, including macrophages, neutrophils, eosinophils, mast cells, and NK cells [48,49]. The surfaces of these immune cells express pathogen-associated molecular patterns (PAMP) and pattern recognition receptors (PRR), which interact with damage-related molecular patterns (DAMP) on damaged cells [50]. When PAMP or DAMP binds to PRR, immune cells activate the signal transduction pathway that changes the expression of transcription factors inside the immune cell, and as a result, immune cells promote immune responses such as cytokines and chemokines secretion, inflammatory response induction, and anti-microbial action [51]. In Fig. 2, Fig. 3, our results show that CP, an immunosuppressant, decreased the CBC (such as WBC, granulocytes, lymphocytes and mid-size cells), level of cytokines (IL-2, IFN-γ, and TNF-α) and IgG in whole blood and serum. The intake of *CGJ*s increased the distribution of immune cells, and the level of cytokines and IgG in whole blood and serum. These results were explained that *CGJ*s regulates the distribution of WBC, granulocytes and lymphocytes, and stimulates immune-related cells to induce the secretion of cytokines cytokines (IL-2, IFN-γ, and TNF-α) and IgG. And it is explained that the intake of *CGJ*s can help increase innate immunity. Additionally, in the inflammatory response, the transcription factors NF-κB and MAPKs (such as JNK, ERK, and p38) are involved in aspects of immune function and acted as pivotal mediators. They induce the expression of pro-inflammatory genes, including those encoding cytokines and chemokines, and participate in inflammatory regulation [50,52]. In Fig. 5, our finding shows that phosphorylation of NF-κB and MAPKs were inhibited in spleen from CP-treated immunosuppressed rat and normalized by the intake of *CGJ*s (S1–S4).

Organ of the immune, spleen is essential for the immune system's function. It filters and removes bacteria and other infectious organisms in the blood. It produces immunoglobulins and have matured NK cells [53]. Therefore, the damage to the spleen is have an increased risk of developing severe bacterial infections. Our results show that the intake of *CGJ*s increased NK cell activity and proliferation and maintained shape of the histological analysis in the spleen (Fig. 4, Fig. 6). Increased NK cell activity in the spleen suggests increase of the ability to respond to cancer cells. Therefore, it is explained that the intake of *CGJ* can also increase the anti-cancer effect.

In conclusion, our finding shows that transitional *CGJ*s made in three different regions and a commercial *CGJ* have immune-enhancing effect. It induced the secretion of cytokines and IgG secretion, changed the gut microbiota and enhancing spleen function. These results suggest that *CGJ* is food that can boost immunity during chemical immunosuppression. However, other factors are also involved in boosting immunity. Our study investigated changes in gut microbiota, but we also need to investigate the effect of its intake on intestinal immunity.

## Data availability statement

Data will be made available on request.

## Funding

This research was supported by “Functional research of fermented soybean food (safety monitoring)” under the Ministry of Agriculture, Food, and Rural Affairs, and partly 10.13039/100030865Korea Agro-Fisheries and Food Trade Corporation in 2023 and the research grant of the 10.13039/501100002510Kongju National University in 2021.

## Institutional review board statement

Animal experiments reported here were approved by the Institutional Animal Care and Use Committee of INVIVO Co., Ltd. (IV-RB-17-2305-15).

## CRediT authorship contribution statement

**Hak Yong Lee:** Writing – original draft, Investigation. **Young Mi Park:** Validation, Investigation, Data curation. **Dong Yeop Shin:** Investigation, Data curation. **Hai Min Hwang:** Investigation. **Han Na Jeong:** Investigation. **Hyo Yeon Park:** Investigation. **Hee-Jong Yang:** Formal analysis. **Gwang Su Ha:** Formal analysis. **Myeong Seon Ryu:** Formal analysis. **Ji Won Seo:** Formal analysis. **Do-Youn Jeong:** Formal analysis. **Jun Sang Bae:** Formal analysis. **Byeong Soo Kim:** Writing – original draft. **Jae Gon Kim:** Writing – review & editing, Writing – original draft, Investigation.

## Declaration of competing interest

The authors declare that they have no known competing financial interests or personal relationships that could have appeared to influence the work reported in this paper.

The author is an Editorial Board Member/Editor-in-Chief/Associate Editor/Guest Editor for *[Journal name]* and was not involved in the editorial review or the decision to publish this article.
