# Supplementary figures and images for: Immune-enhancing effect of fermented soybean food, Cheonggukjang on cyclophosphamide-treated immunosuppressed rat
Source: Heliyon. 2024 Sep 14;10(18):e37845. doi: 10.1016/j.heliyon.2024.e37845 (PMC11425096; doi:10.1016/j.heliyon.2024.e37845)

Phospho Erk

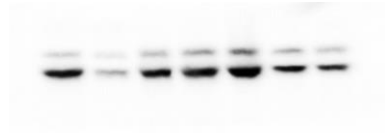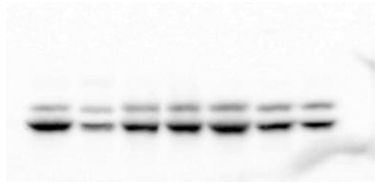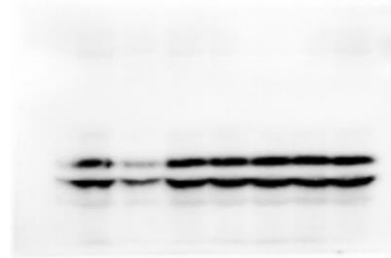

Erk

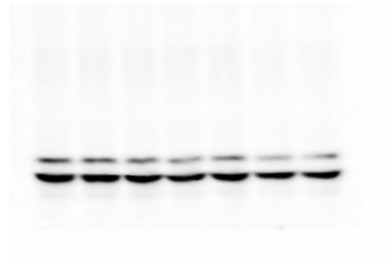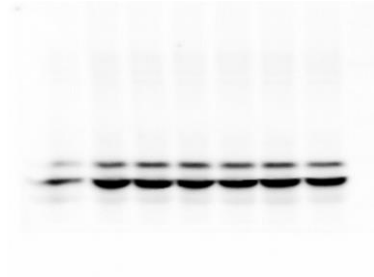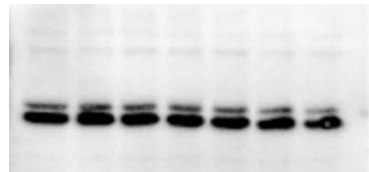

Phospho p38

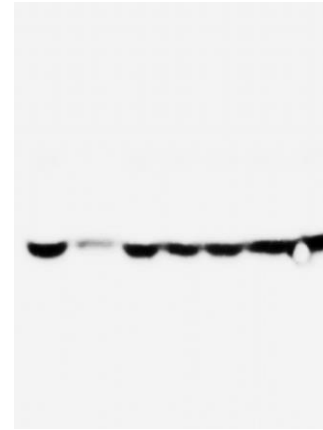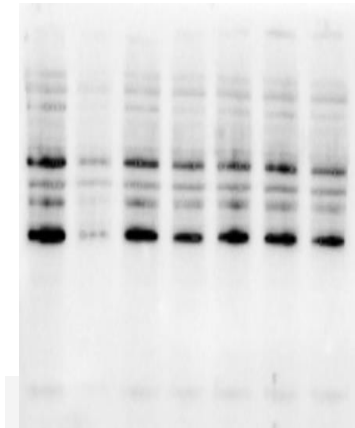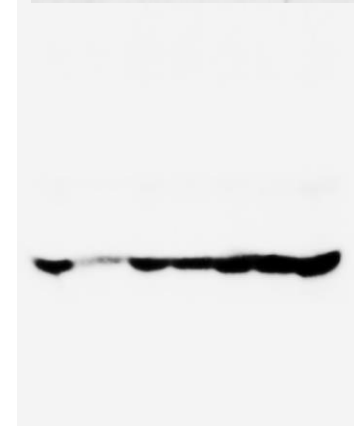

p38

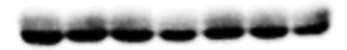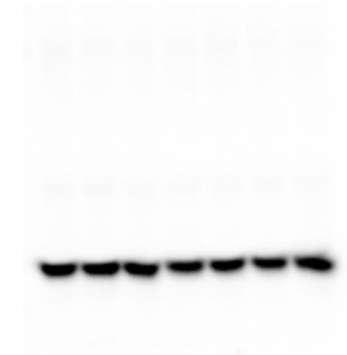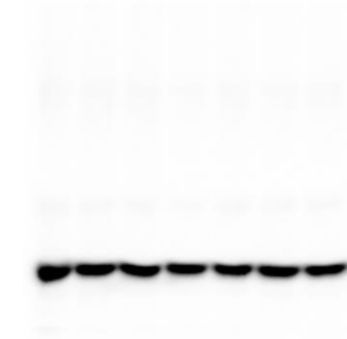

Phospho JNK

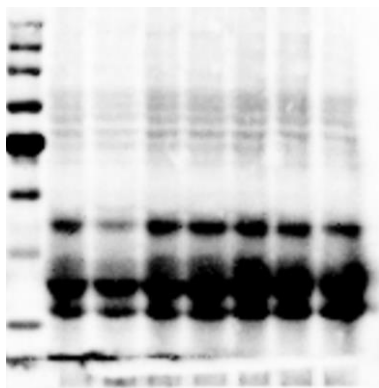

JNK

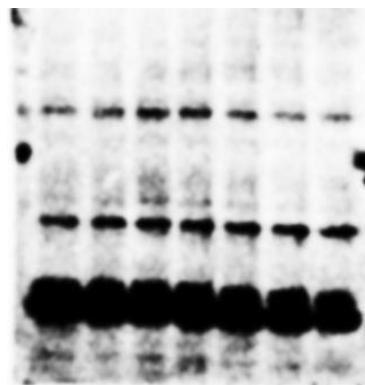

Phospho NFκB

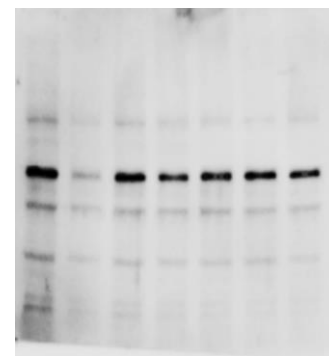NF $\kappa$ B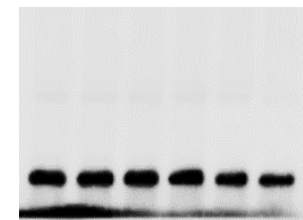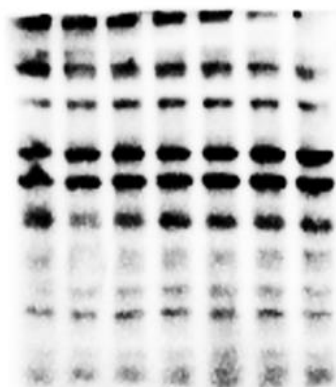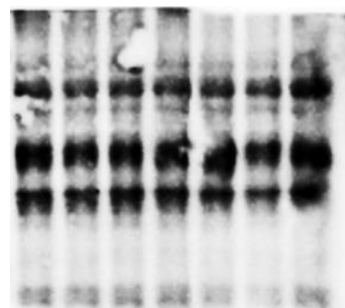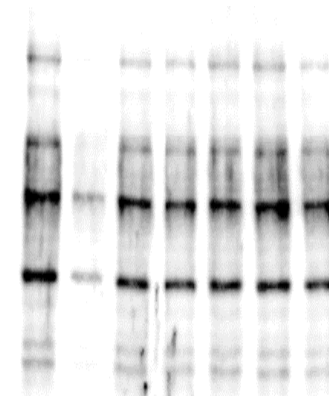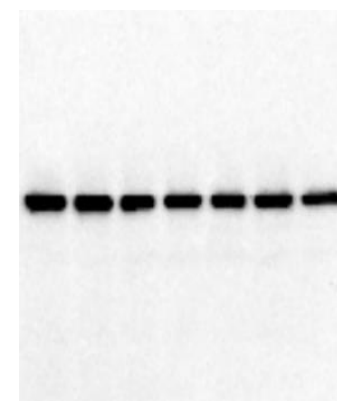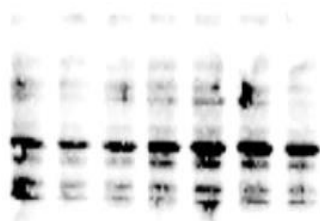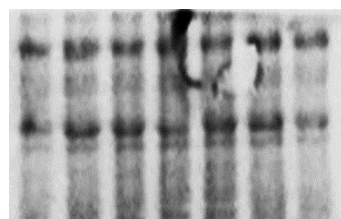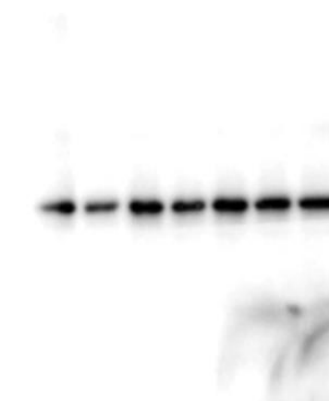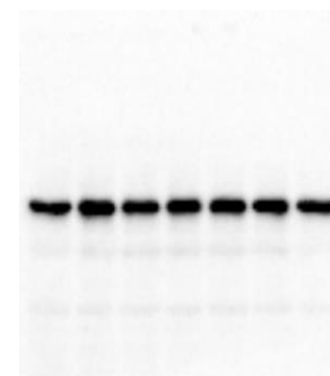

Supplement: Multimedia component 1 [file mmc1.pdf]
